# The Glucose Sensor-Like Protein Hxs1 Is a High-Affinity Glucose Transporter and Required for Virulence in *Cryptococcus neoformans*

**DOI:** 10.1371/journal.pone.0064239

**Published:** 2013-05-14

**Authors:** Tong-Bao Liu, Yina Wang, Gregory M. Baker, Hany Fahmy, Linghuo Jiang, Chaoyang Xue

**Affiliations:** 1 Public Health Research Institute Center, University of Medicine and Dentistry of New Jersey, Newark, New Jersey, United States of America; 2 Department of Microbiology and Molecular Genetics, University of Medicine and Dentistry of New Jersey, Newark, New Jersey, United States of America; 3 Tianjing Medical University, Tianjing, China; 4 The National Engineering Laboratory for Cereal Fermentation Technology, School of Biotechnology, Jiangnan University, Wuxi, China; Yonsei University, Republic of Korea

## Abstract

*Cryptococcus* is a major fungal pathogen that frequently causes systemic infection in patients with compromised immunity. Glucose, an important signal molecule and the preferred carbon source for *Cryptococcus*, plays a critical role in fungal development and virulence. *Cryptococcus* contains more than 50 genes sharing high sequence homology with hexose transporters in *Saccharomyces cerevisiae*. However, there is no report on their function in glucose sensing or transport. In this study, we investigated two hexose transporter-like proteins (Hxs1 and Hxs2) in *Cryptococcus* that share the highest sequence identity with the glucose sensors Snf3 and Rgt2 in *S. cerevisiae*. The expression of *HXS1* is repressed by high glucose, while the *HXS2* expression is not regulated by glucose. Functional studies showed that Hxs1 is required for fungal resistance to oxidative stress and fungal virulence. The *hxs1Δ* mutant exhibited a significant reduction in glucose uptake activity, indicating that Hxs1 is required for glucose uptake. Heterologous expression of *Cryptococcus HXS1* rendered the *S. cerevisiae* mutant lacking all 20 hexose transporters a high glucose uptake activity, demonstrating that Hxs1 functions as a glucose transporter. Heterologous expression of *HXS1* in the *snf3Δ rgt2Δ* double mutant did not complement its growth in YPD medium containing the respiration inhibitor antimycin A, suggesting that Hxs1 may not function as a glucose sensor. Taken together, our results demonstrate that Hxs1 is a high-affinity glucose transporter and required for fungal virulence.

## Introduction

The ability of a pathogen to sense extracellular signals and adapt to the host environment is essential for the establishment of an infection during a host-pathogen interaction. Characterization of extracellular signals and their sensors in a pathogen is central for understanding its pathogenesis. *Cryptococcus neoformans* is a major human fungal pathogen and the causative agent of the often fatal cryptococcal meningoencephalitis, which is an AIDS-defining illness [1]. *Cryptococcus*, a haploid yeast pathogen, is an ideal model system to study signal transduction in pathogenic fungi. Several signaling pathways important for *Cryptococcus* virulence have been identified [2], [3], [4], [5]. However, extracellular signals and their sensors remain largely unknown.

Glucose is the preferred carbon source for yeasts, including *C. neoformans*. It also functions as a hormone-like signal molecule for the regulation of cellular function and glucose utilization in *Saccharomyces cerevisiae* [6]. In *C. neoformans*, glucose sensing and utilization is required for fungal virulence. Host macrophages are the first line of host defense mechanism against cryptococcal infection. The antiphagocytic protein App1 is a fungal virulence factor and inhibits macrophage-mediated phagocytosis via complement receptor 3 (CR3) [7]. Expression of App1 is highly induced during lung infection due to the low glucose concentration (∼ 0.002%) environment in the lung and macrophages [8]. These results demonstrate the importance of glucose as a host signal in regulation of *Cryptococcus*-macrophage interactions [8]. A fully functional glycolytic pathway for proper glucose utilization is critical for cryptococcal infection and persistence of the fungus in the central nervous system (CNS) [9], [10]. UDP-glucuronic acid as a product of glycolysis is involved in the formation of the extracellular polysaccharide capsule, a major virulence factor [11], [12]. Despite the importance of glucose, it remains unknown how glucose is sensed and how glucose acquisition is regulated in *C. neoformans*. It has been shown that one G protein-mediated signaling pathway, the Gpa1-cAMP pathway, can be activated by glucose in *C. neoformans* and plays a central role in fungal virulence [13], [14]. Glucose can no longer activate the cAMP signaling in a *gpa1Δ* mutant background, indicating the Gpa1 G protein is essential for glucose signaling [15], [16]. However, the cell surface receptor that senses glucose to activate Gpa1 remains to be identified. One possibility is the G protein-coupled receptor (GPCR) family members function as glucose receptors to sense glucose and activate cAMP signaling via Gpa1. We have identified three GPCR proteins (Gpr4, Gpr5, and Ste3**a**) that are involved in the Gpa1 signaling activation, but none of them is required for glucose sensing [15], [17]. Alternatively, other mechanisms may be involved in the cAMP signaling activation.

Glucose acquisition and utilization systems have been extensively studied in *S. cerevisiae*, which employs two glucose sensory systems to sense the availability of extracellular glucose and fine-tune the function of a large gene family of hexose transporters as glucose carriers [6], [18], [19], [20]. One GPCR, Gpr1, functions as a glucose sensor to activate a downstream G protein signaling pathway, the Gpa2-cAMP pathway [21], [22], which is parallel to the Gpa1-cAMP pathway in *C. neoformans* and is required for cell growth and metabolic activity regulation in response to the availability of nutrients. Besides Gpr1, two unusual members of the hexose transporter gene family, Snf3 and Rgt2, can also sense different levels of extracellular glucose. Snf3 and Rgt2 maintain hexose transporter structures and possess unique long C-terminal cytoplasmic tails. However, they cannot transport glucose, and instead function as glucose sensors. Snf3 senses low glucose concentrations and activates high affinity hexose transporters, while Rgt2 senses high levels of glucose to regulate the expression of low affinity transporters [23]. These two sensors can interact with the casein kinase I Yck1/2, which are responsible for phosphorylation of Mth1 and Std1, two transcriptional regulators [24]. Mth1 and Std1 form a complex with another regulator Rgt1 to repress the expression of hexose transporters [19]. Phosphorylated Mth1 and Std1 are subjected for ubiquitination and degradation through an SCF(Grr1) E3 ligase-mediated ubiquitin-proteasome pathway. The degradation of Mth1 and Std1 in the 26S proteasome releases the binding of Rgt1 on promoters of hexose transporters, which in turn activates the expression of a number of hexose transporters for glucose uptake [25].

Besides Rgt2 and Snf3 in *S. cerevisiae,* glucose sensors have also been identified in the pathogenic yeast *Candida albicans* (Hgt4) [26] and in the methylotrophic yeast *Hansenula polymorpha* (Hxs1) [27]. *C. neoformans* also contains a large group of hexose transporter homologs based on available genome sequences, but how these transporters function in response to glucose availability is unknown. In this study, we identified two hexose transporter candidates (Hxs1 and Hxs2) that share the highest sequence identity with Snf3 and Rgt2 in *S. cerevisiae*. Hxs1 expression is repressed by glucose, while Hxs2 expression is not regulated by glucose. Functional studies in *C. neoformans* demonstrate that Hxs1 is required for oxidative stress response and fungal virulence. Heterologous expression of *HXS1* in a *Saccharomyces* strain lacking all hexose transporters showed high glucose uptake activity. Expressing *HXS1* in the *snf3Δ rgt2Δ* double mutant failed to complement the regulatory function of these sensors. Our results indicate Hxs1 is a high-affinity glucose transporter rather than a glucose sensor.

## Results

### Hxs1 and Hxs2 are Homologs of Snf3 and Rgt2 in *S. cerevisiae*

**Figure 1:**
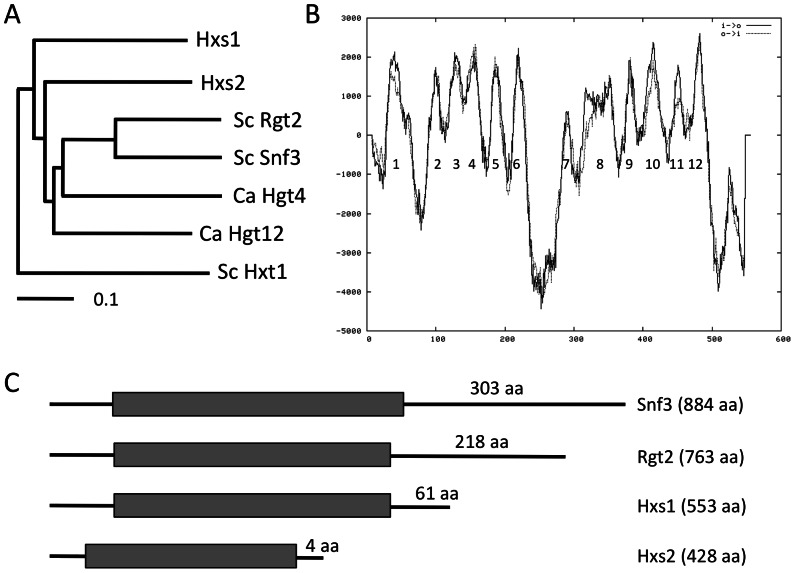
Identification of Snf3/Rgt2 homologs in *Cryptococcus*. **A.** Phylogenetic tree of proteins from *C. albicans* (Hgt4 and Hgt12) and *C. neoformans* (Hxs1 and Hxs2) that share high homology with glucose sensors Snf3 and Rgt2 in *S. cerevisiae*. The full-length protein sequences were used for alignment using ClustalX. The hexose transporter Hxt1 in *S. cerevisiae* was used as an out-group. **B.** Predicted topology of the deduced Hxs1 amino acid sequence based on the TMpred program. Hydropathy values are on the y-axis, and the residue numbers are on the x-axis. The predicted transmembrane domains (TM1 to 12) are numbered. **C.** Schematic models of the primary protein structures of Snf3, Rgt2, Hxs1 and Hxs2. The black boxes represent transmembrane regions. The total number of amino acids for each protein and its C-terminal tail are indicated.

Based on the genome sequence of H99 strain, *C. neoformans* contains a large gene family of hexose transporter homologs with around 55 members. However, there is no report about their function in glucose sensing or transport. We compared the hexose transporter gene family members among *S. cerevisiae*, *Candida albicans*, and *C. neoformans*, and identified a cluster of proteins showing high sequence similarity with the glucose sensors Snf3 and Rgt2 in *S. cerevisiae* (Fig. S1 and Fig. 1A). We named two *Cryptococcus* proteins in this cluster as hexose sensor-like protein 1 and 2 (Hxs1 and Hxs2). Hxs1 contains 12 transmembrane domains, a typical structure of hexose transporters (Fig. 1B). Both Hxs1 (CNAG_03772; 553 amino acids) and Hxs2 (CNAG_04931; 428 amino acids) are much smaller than Snf3 or Rgt2 in *S. cerevisiae.* Interestingly, neither of them has the long C-terminal tail and the conserved C-terminal domains (R1 and R2) that exist in glucose sensors, including Snf3 and Rgt2 (Fig. 1C) [26], [27].

### *HXS1* Expression is Repressed by Glucose

**Figure 2:**
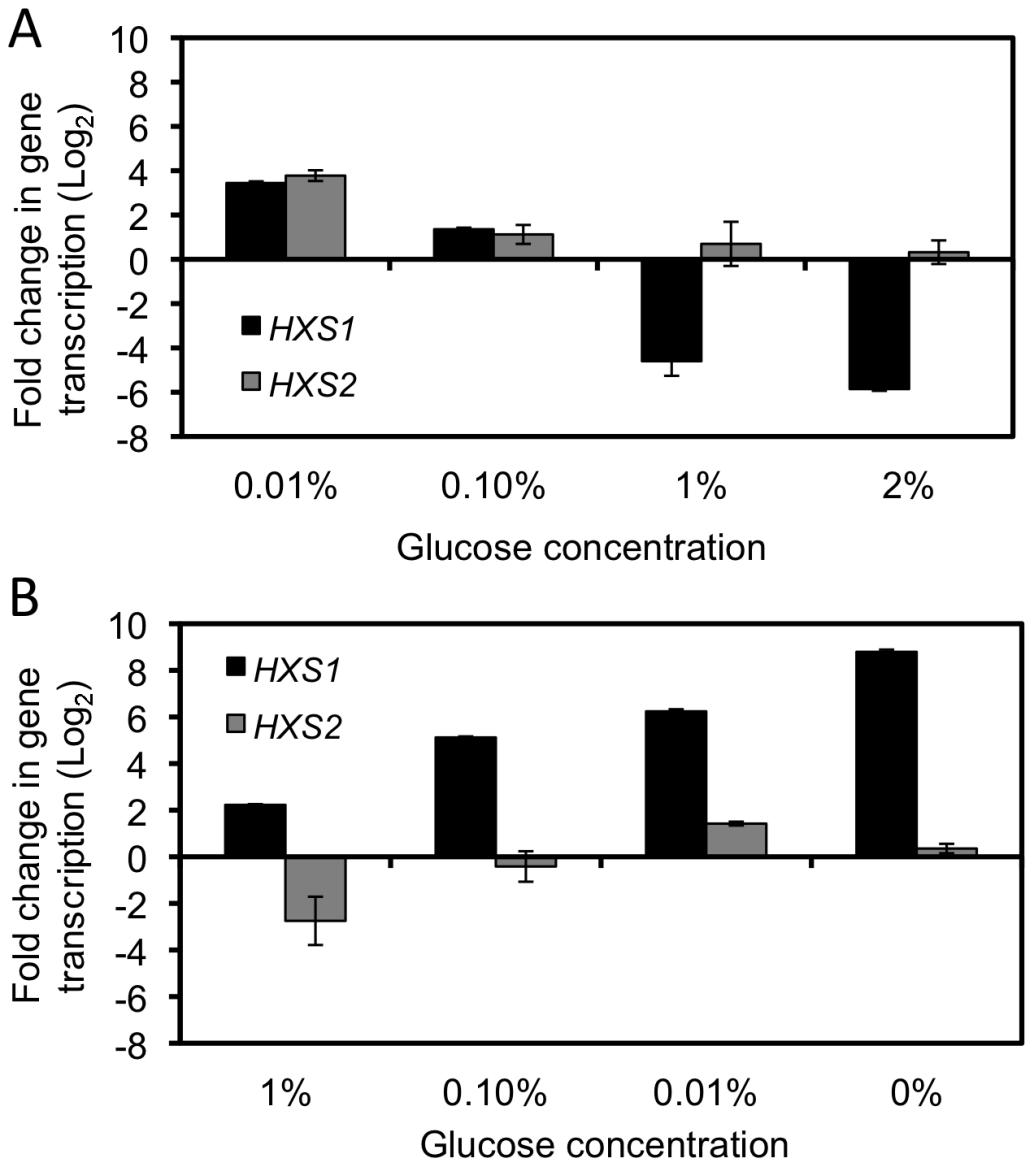
The expression of *HXS1* is repressed by glucose. The qRT-PCR method was used to detect the change in transcription levels of *HXS1* and *HXS2* under growth conditions with different glucose levels. H99 cells were grown on YPG (no glucose) liquid medium, then switched to medium with 0.01%, 0.1%, 1% or 2% glucose (**A**), or grown on YPD and switched to medium with lower glucose levels (1%, 0.1%, 0.01% or YPG) (**B**). Cells were collected after 2 hr incubation and RNA prepared for qRT-PCR. Values are expressed as relative expression (log2) of the *HXS1* or *HXS2* gene, normalized to the *GAPDH* gene endogenous reference. The changes in gene transcription levels were related to 0-hr time point (H99 overnight liquid cultures on either YPG (**A**) or YPD (**B**)). The error bars showed standard deviations of three repeats.

To understand the role of Hxs1 and Hxs2 in glucose utilization, we measured the transcriptional regulation of both Hxs1 and Hxs2 under conditions with different levels of glucose. The culture of the wild type strain H99 growing on medium without glucose but containing 2% galactose (YPG) was switched to medium with 0.01%, 0.1%, 1% or 2% glucose and incubated for 2 hrs, and the expression of *HXS1* was measured at transcription level by qRT-PCR (Fig. 2). Our results showed a pattern of decreased *HXS1* expression following the increase of glucose concentrations in the medium. When the glucose concentration was 1% or higher, the transcription level of *HXS1* decreased significantly (>16 fold) (Fig. 2A). In contrast, when the H99 culture on YPD (2% glucose) was switched to media with lower glucose concentrations (1%, 0.1%, or 0.01%), an significant increase of *HXS1* transcription levels was observed (>4 fold) (Fig. 2B). These results showed that the expression of *HXS1* is dramatically repressed by the presence of high glucose levels. On the other hand, the expression of *HXS2* is very low and is not regulated by glucose concentrations (Fig. 2). We hardly detected the PCR signal of *HXS2* even after 35 cycles of amplification (Fig. S2).

### Hxs1 does not Regulate the Expression of *HXT*s in *C. neoformans*

We then generated *hxs1Δ* mutants by homologous recombination to examine the role of Hxs1 in the regulation of glucose utilization in *C. neoformans*. We failed to generate the gene deletion mutant for the *HXS2* gene, which may be due to its telomere location in the genome. The *HXS2* gene is the first gene in Chromosome 10 with only 1098 bp away from the beginning of the chromosome, which likely prevented the homologous recombination event from happening to replace the *HXS2* gene with a marker. Hence, we focus on the analysis of Hxs1 in this study.

**Figure 3:**
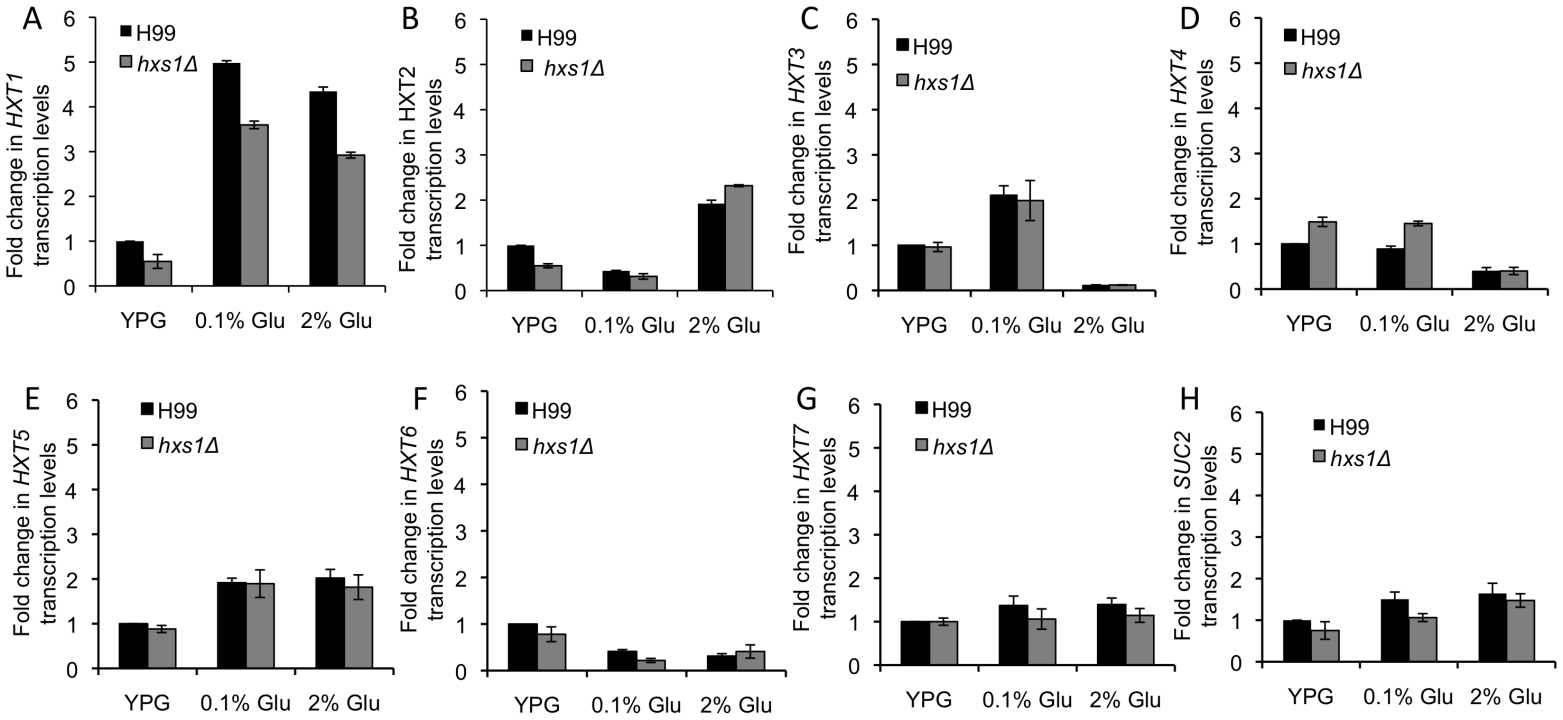
Hxs1 is not required for the expression of other hexose transporter homologs. The qRT-PCR method was used to measure the expression of seven hexose transporter homologs *HXT1-7* (A–G) and *SUC2* (H) in *C. neoformans* under YPG, YP with 0.1% glucose, or YPD (2% glucose) growth conditions. Values are expressed as relative expression of these genes, normalized to the *GAPDH* gene endogenous reference, and relative to *HXS1* expression in H99 on YPG medium. Error bar indicates the standard deviation of three repeats.

In *S. cerevisiae*, Hxt1 is a low-affinity glucose transporter, while Hxt2 is a high-affinity glucose transporter. The expression of *HXT1* is induced only by high glucose concentration, while *HXT2* is induced only by low glucose levels [23]. We compared the expression of *Cryptococcus HXT1* and *HXT2* homologs in the wild type and the *hxs1Δ* mutant under different glucose conditions. Because there are multiple protein homologs sharing high sequence identities with Hxt1 and Hxt2 in *S. cerevisiae* (E score is 0), we selected the first seven hits and investigated their expressions under different glucose conditions. Our qRT-PCR results showed that two gene (CNAG_03438, *HXT1;* CNAG_04920, *HXT5*) were induced by both low and high glucose concentrations, with a significantly higher induction in *HXT1*. Meanwhile, *HXT2* (CNAG_06290) was induced only by the high glucose concentration, while *HXT3* (CNAG_05387) was induced only by low glucose condition (Fig. 3). The changes of transcription level of other tested *HXT* homologs, *HXT4* (CNAG_06521), *HXT6* (CNAG_06963) and *HXT7* (CNAG_03432), were not significant (<2 fold). The transcription level of each *HXT* gene tested was not altered significantly by the lack of *HXS1* (<2 fold) when compared to the wild type H99. The changes of *HXT1* transcription level between in H99 and the *hxs1Δ* mutant backgrounds were ∼1.4 folds on both glucose conditions, but was still significantly induced by glucose in both backgrounds. Hence, our results suggest the expression of all seven *HXT* gene homologs in *C. neoformans* is independent of Hxs1 (Fig. 3A–G).

In *S. cerevisiae*, the expression of hexose transporters was regulated by the Glucose sensors Rgt2 and Snf3 via the SCF(Grr1) E3 ligase [25]. We have identified an F-box protein, Fbp1, as the Grr1 homolog in *C. neoformans* [28]. To investigate the potential role of Hxs1 in the regulation of glucose uptake, we examined the expression of *FBP1,* as well as *CCK1*, a gene encoding the casein kinase, under low and high glucose conditions in both H99 and *hxs1Δ* backgrounds. Our qRT-PCR results showed that Hxs1 is not required for the regulation of these two genes in both low and high glucose conditions (data not shown). Because the expression of *SUC2*, a gene encoding sucrose invertase, is repressed by high glucose in *S. cerevisiae* and has been used as an indicator for the glucose repression [29], we also measured the expression of *SUC2* homolog in *C. neoformans* using qRT-PCR. Interestingly, the *SUC2* homolog in *C. neoformans* was not repressed by glucose. Hence, our results showed that the expression of this gene was not regulated by Hxs1 either (Fig. 3H). It is possible that the *Suc2* protein has either different function or the glucose regulatory mechanism in *C. neoformans* is different from that of *S. cerevisiae*.

### Hxs1 is Required for Efficient Glucose Uptake and Growth on Low Glucose Conditions

**Figure 4:**
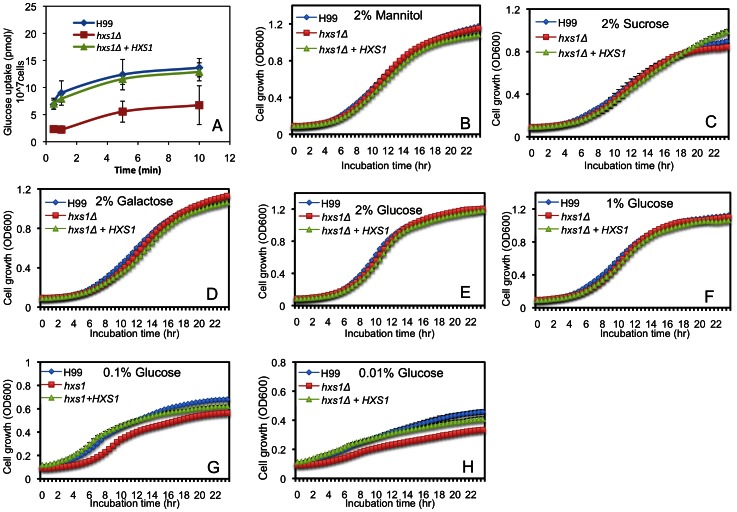
Hxs1 is required for *Cryptococcus* glucose uptake and cell growth on low glucose media. **A.** **** Glucose uptake assay was performed for *Cryptococcus* wild type H99, the *hxs1Δ* mutant or its complemented strain as described in Materials and Methods. Error bar indicates the standard deviation of three repeats. **B–H**. Cryptococcus cell growth was assayed in 96-well plates. 1×10^5^ cells of each strain were inoculated into the wells containing 100 µl YP supplemented with either 2% mannitol (**B**), 2% sucrose (**C**), 2% galactose (**D**), 2% glucose (**E**), 1% glucose (**F**), 0.1% glucose (**G**), or 0.01% glucose (**H**). The plates were kept in a PerkinElmer precisely Envision 2014 Multilabel Reader and incubated at 30°C with shaking (350RPM) and OD600 were measured in real time every half hour. Each experiment was performed in triplicates. Error bars indicate standard deviations.

To investigate the potential role of Hxs1 in *Cryptococcus* glucose uptake, ^3^H-labeled glucose uptake assays were performed in the wild type, the *hxs1Δ* mutant and its complemented strain. Our results showed that the *hxs1Δ* mutant had significantly lower glucose uptake activity than the wild type or the complemented strain, suggesting that Hxs1 plays a major role in glucose uptake (Fig. 4A).

Glucose is the preferred carbon source for *Cryptococcus*, and also plays an important role in the development of virulence factors. Because Hxs1 is important for glucose uptake, we tested the effect of Hxs1 on the growth of *Cryptococcus* cells on media with different glucose levels (2%, 1%, 0.1% or 0.001%). We also examined the growth of mutant cells on media with different carbon sources (mannitol, sucrose or galactose). Our results showed that when cells were grown on media with high levels of glucose or other tested carbon source, no growth defect was observed (Fig. 4B–E). However, on media with low glucose levels, the *hxs1Δ* mutant showed a small but significant growth defect (Fig. 4F–H), indicating that Hxs1 is important for the fungus to survive under conditions with low glucose availability.

### Hxs1 is Required for Cell Integrity and Stress Response

**Figure 5:**
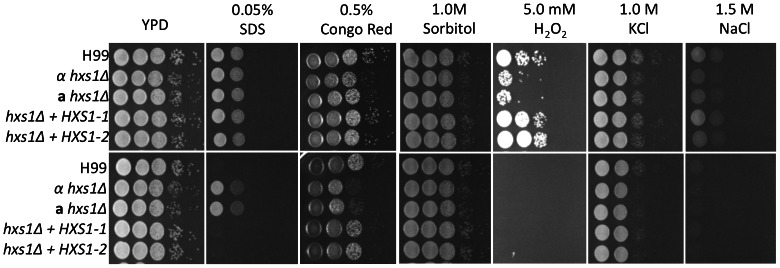
Hxs1 is required for stress response. Cultures of wild type H99, the *hxs1Δ* mutant and its complemented strain were inoculated on YPD with 0.05% SDS, 0.5% Congo Red, 1 M Sorbitol, 5 mM H_2_O_2_, 1 M KCl, or 1.5 M NaCl, respectively. Plates were incubated at 30°C (upper) or 37°C (lower) for 3 days and photographed.

To address the cellular function of Hxs1, we analyzed the potential phenotype of the *hxs1Δ* mutant under different stress conditions. Phenotypic analyses showed that the *hxs1Δ* mutant had normal growth on medium with high salt or high osmolarity, indicating that Hxs1 is not required for these stress resistance conditions. However, the *hxs1Δ* mutant had a growth defect on medium with 5 mM H_2_O_2_, indicating Hxs1 is involved in cell resistance to oxidative stress. Interestingly, the *hxs1Δ* mutant showed a better growth at higher temperature (37°C) on medium with SDS, suggesting it is more resistant to SDS treatment (Fig. 5).

**Figure 6:**
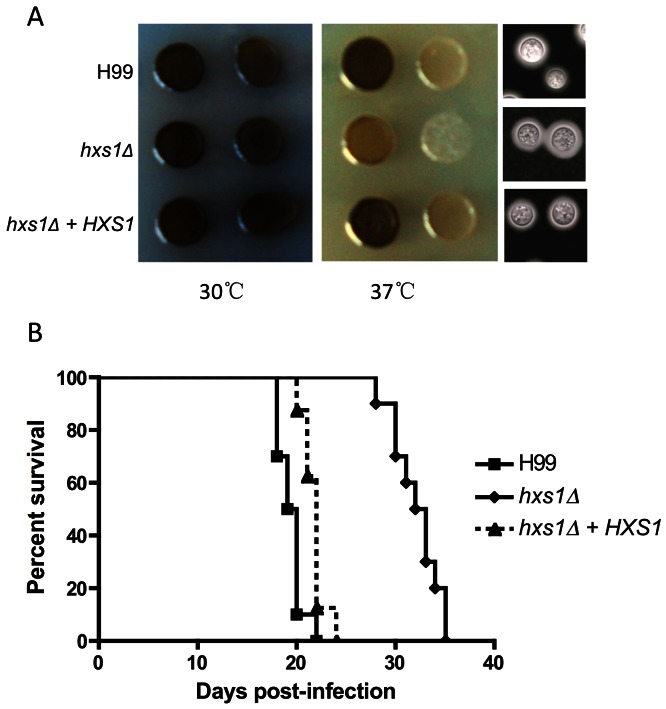
Hxs1 is required for fungal virulence. **A.** Cultures of H99, *hxs1Δ* and its complement strain were inoculated on DME medium for capsule induction, or on L-DOPA medium for melanin induction. Plates were incubated at 30°C or 37°C for 48 hr and photographed. **B.** To determine the fungal virulence, female A/Jcr mice were intranasally infected with 10^5^ cells of H99, *hxs1Δ* and its complement strain. Animals were monitored for clinical signs of cryptococcal infection and sacrificed at predetermined clinical endpoint that predicts imminent mortality.

Because glucose sensing regulates capsule and melanin production via the cAMP signaling pathway [2], [15], we also examined the potential involvement of Hxs1 in the development of virulence factors. Our studies showed that the *hxs1Δ* mutant produced normal melanin on L-DOPA medium at 30°C, but had a modest melanin defect at 37°C. No obvious difference in capsule production was observed between the wild type and the mutant (Fig. 6A).

### Hxs1 is Required for Fungal Virulence

Because the importance of glucose for fungal cellular development and the importance of Hxs1 in stress response and melanin production, we examined the potential impact of Hxs1 on fungal virulence using a murine inhalation infection model of cryptococcosis. In accord with previous results [28], all mice infected with 10^5^ yeast cells of wild-type strain H99 had a median survival time of 19.5 days due to lethal infection. In contrast, the *hxs1Δ* mutant showed significant virulence attenuation (P<0.0001) with a median survival time of 32.5 days (Fig. 6B). This result demonstrates that Hxs1 is required for fungal full virulence.

### Hxs1 Showed High-affinity Uptake Activity in a Heterologous Expression System

**Figure 7:**
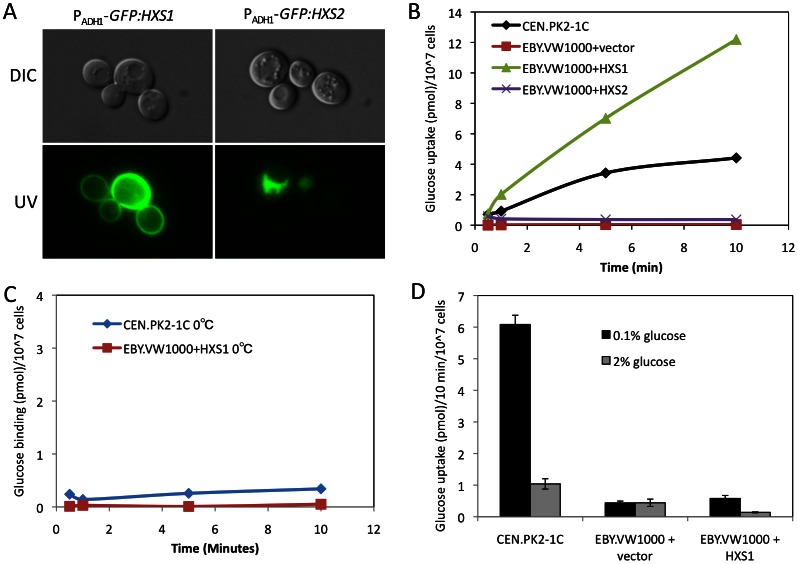
Heterologous expression of *HXS1* in a *Saccharomyces* strain lacking hexose transporters showed high glucose uptake activity. **A.** Localization of *HXS1*, *HXS2* in *S. cerevisiae* was determined by overexpressing a *GFP:HXS1* or *GFP:HXS2* fusion protein in EBY.VW1000, a mutant lacking all hexose transporters. EBY.VW1000 expressing an empty vector was used as a control. **B and C.** Glucose uptake activity (**B**) or glucose binding activity (C) of a *Saccharomyces* strain expressing *HXS1*. Cultures of the background strain CEN.PK2.1C and EBY.VW1000 expressing the pTH74 empty vector, the *HXS1*, or the *HXS2* genes were inoculated on SD media lacking Uracil but containing 2% maltose as carbon source. Yeast cells were mixed with ^3^H-labeled glucose and incubated at 30°C(**B**) or 0°C (**C**) for 1, 5, and 10 mins. This assay was repeated twice with similar patterns. **D.** Glucose uptake assay was performed for *S. cerevisiae* strains CEN.PK2.1C and EBY.VW1000 expressing the empty vector or *HXS1* in the presence of 0.1% or 2% cold glucose. The error bar indicates the standard deviation of three repeats.

To examine whether Hxs1 and Hxs2 in *C. neoformans* function as glucose sensors or transporters, *GFP:HXS1* and *GFP:HXS2* fusion constructs were expressed under the control of the *ADH1* promoter in an *S. cerevisiae* mutant strain EBY.VW1000, in which all 20 hexose transporters are deleted [30]. The EBY.VW1000 strain thus cannot grow on YPD medium. The expression of *HXS1* was confirmed by GFP signals and RT-PCR. Our results showed that GFP:Hxs1 was localized on cell plasma membrane as expected (Fig. 7A).

Glucose uptake assay was performed to examine the uptake ability of Hxs1 in this heterologous expression strain. We used 1 µCi ^3^H-glucose for all assays and found that the strain expressing GFP:Hxs1 could transport glucose at a rate much higher than that of the wild type strain, likely due to its overexpression. The wild type strain becomes saturated after 5 mins, while the *HXS1*-expressing strain remains efficient uptake of ^3^H-labeled glucose, an indication of impaired regulation on glucose uptake (Fig. 7B). To examine the possibility that the outcome of uptake assays were resulted from glucose binding, instead of transport, we also performed the glucose binding assay at 0°C, and found that each strain only can bind to very limited amount of glucose (Fig. 7C). We also performed glucose uptake assays by addition of cold glucose to compete with the ^3^H-labeled glucose. In the presence of 0.1% cold glucose, the wild type strain still showed high uptake of labeled glucose, while its uptake signal was significantly reduced when 2% glucose was added in the reaction. In contrast, very low uptake signal was detected in the strain expressing *HXS1*, by adding either 0.1% or 2% cold glucose (Fig. 7D). Overall, these results demonstrate that Hxs1 is a high-affinity glucose transporter.

We also introduced the *GFP:HXS2* overexpression construct into the same strain. To our surprise, even though the construct was prepared exactly same as that of the *GFP:HXS1* construct with correct sequence, the GFP signal of the strain expressing GFP:Hxs2 was very weak. For a small population of cells that showed stronger signal, most of the fluorescence signal was localized in the vacuole instead of cell plasma membrane (Fig. 7A). It remains unclear whether the GFP:Hxs2 fusion protein is functional. We also repeated the glucose uptake assay for this strain and found the strain expressing GFP:Hxs2 fusion protein failed to transport glucose, suggesting the Hxs2 protein might not function in glucose uptake (Fig. 7B).

### Hxs1 could not Rescue the Growth Defect of the *snf3Δ rgt2Δ* Double Mutant on YPD with Antimycin A

**Figure 8:**
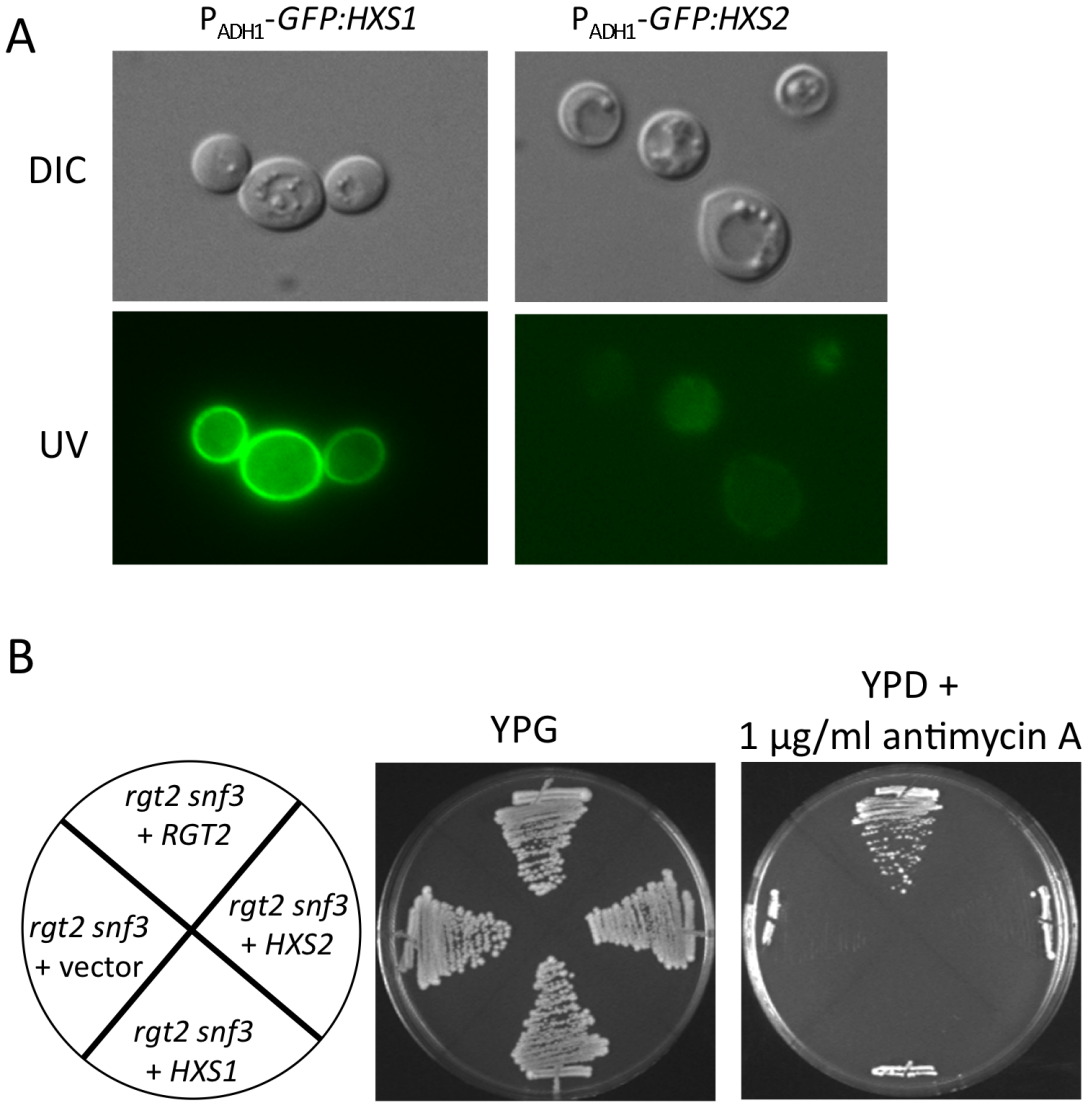
Heterologous expression of *HXS1* in the *snf3Δ rgt2Δ* double mutant background failed to complement glucose sensor function. **A.** Localization of *HXS1*, *HXS2* in *S. cerevisiae* was determined by overexpressing a *GFP:HXS1*, *GFP:HXS2, or GFP:RGT2* fusion protein in a *Saccharomyces* strain lacking both Rgt2 and Snf3 glucose sensors. **B.** The growth of *S. cerevisiae* wild type BY4741, the *snf3Δ rgt2Δ* double mutant expressing empty vector pTH74, *HXS1*, *HXS2*, and *RGT2* were inoculated on YPG or YPD with 1 µg/ml antimycin A. Plates were incubated at 30°C for 3 days.

To further investigate the possibility that Hxs1 and Hxs2 may function as glucose sensors, we generated a *S. cerevisiae snf3Δ rgt2Δ* double mutant by genetic crossing of a *snf3Δ* mutant and a *rgt2Δ* mutant (kindly provided by Dr. Mark Johnston). The *pADH1*-*GFP:HXS1* and *pADH1*-*GFP:HXS2* constructs were introduced in this *snf3Δ rgt2Δ* double mutant, respectively. Their expressions were confirmed by GFP signals (Fig. 8A). While a strong GFP signal was observed for strains expressing *HXS1*, only a weak fluorescent signal was observed for strains expressing *HXS2*, which is consistent with their expression in EBY.VW1000, the strain lacking all hexose transporters (Fig. 7A). Based on previous studies, the double mutant could not grow on YPD medium containing 1 µg/ml antimycin A, which substantially inhibits respiration by blocking electron transfer between cytochromes b and c [26], [31]. We thus tested the growth of these strains expressing *HXS1* or *HXS2* on YPD medium with 1 µg/ml antimycin A. Consistent with the previous report, the double mutant expressing an empty vector could not grow on this medium, while reintroducing a *RGT2* copy rescued the growth defect. However, the double mutant expressing either *HXS1* or *HXS2* failed to grow on this medium, indicating that neither of them could complement the function of glucose sensors (Fig. 8). Thus, Hxs1 and Hxs2 may not function as glucose sensors. It is likely that Hxs2 is not a functional protein.

Overall, our results showed that Hxs1 is a high-affinity glucose transporter. We have seen no activity of Hxs2 and it is possible that Hxs2 may not function properly in *Cryptococcus*. Other hexose transporter-like proteins may function as glucose sensors to regulate glucose uptake.

## Discussion

Studies in *S. cerevisiae* revealed a complex network of glucose sensing, regulation of glucose uptakes, and subsequent glucose utilization. There are two major glucose sensory systems in the baker’s yeast, Rgt2/Snf3 and Gpr1, that coordinate the function of the large hexose transporter gene family for optimized glucose utilization [19]. In *C. neoformans*, how the fungus senses and acquires glucose remains unclear. The genome of *Cryptococcus* revealed a large gene family with over 50 members that shares high sequence identity with hexose transporters in other yeasts. There is only one previous report that attempted to link one hexose transporter homolog to copper resistance in *C. neoformans* [32]. How these genes are regulated and whether there is a similar transporter-like glucose sensor remains unknown. The identification of Hxs1 and Hxs2 in this report is the first attempt to study the function of this important gene family in glucose utilization.

Based on the phylogenetic relationship we presented in Figure S1, Hxs1 and Hxs2 have the highest sequence identity with the Rgt2 and Snf3 in *S. cerevisiae,* also with Hgt4, a glucose sensor in *C. albicans* [26]. However, neither Hxs1 nor Hxs2 has the long C-terminal tail or the conserved R1 or R2 domains in that region that has been shown important for the sensory function in other glucose sensors. There was no hit when we searched the H99 genome database with either R1 or R2 domains from Snf3 protein sequence, so whether such sequences exist in *C. neoformans* remain unknown. Hxs1 is repressed by glucose while Hxs2 is very weakly expressed and not regulated by glucose. Our mutagenesis studies showed that Hxs1 plays an important role in fungal virulence. Uptake assays for the *hxs1Δ* mutant and a *S. cerevisiae* mutant strain expressing *HXS1* demonstrated that Hxs1 is required for glucose transport. Human and animal lungs only contains very low level of glucose [8]. To survive in such an environment, an efficient glucose uptake activity could be important for the pathogen, which may explain why the *hxs1Δ* mutant showed a significant virulence attenuation. The finding that Hxs1 is a high-affinity glucose transporter further explains its importance during *Cryptococcus*-host interaction, especially in an environment where glucose is scarce. In addition, we also observed that the *hxs1Δ* mutant had a defect in melanin production, a major virulence factor, and cell resistance to oxidative stress, which may also contribute to its defect in virulence.

Because we still could not generate a *hxs2Δ* null mutant even after many repeats, the function of Hxs2 remains to be determined. It is possible that Hxs2 is not functional due to its telomere location. *HXS2* is located at the beginning of the chromosome 10, which could be the reason why we could not delete the *HXS2* gene. Genes located in the telomere region frequently undergo gene duplication, rearrangement, and transcriptional silencing [33], [34], [35]. Although remaining to be proven, we suspect that *HXS2* could be silenced due to the telomere-associated position effect and thus not functional. Although we could amplify the cDNA sequence of *HXS2*, its amplification was not robust may due to its extreme low expression or silencing effect.

Our heterologous expression assays using the *S. cerevisiae* strain lacking all hexose transporters showed that Hxs1 functions as a high-affinity transporter. When *HXS1* was expressed in an *S. cerevisiae* mutant lacking both glucose sensors, it did not rescue the growth defect on YPD medium with antimycin A, suggesting that Hxs1 may not have the property of glucose sensing. Thus, whether *Cryptococcus* has a Rgt2/Snf3-like glucose sensory system remains to be determined. Although our data does not suggest that Hxs1 or Hxs2 plays a role in glucose sensing, we could not completely rule out the possibility that Hxs1 may be a dual function transporter, functioning as both a transporter and a sensor, similar to the ammonium transporter Mep2 [36]. Because there are over 50 hexose transporter-like proteins, although Hxs1 and Hxs2 share the highest sequence similarity with Snf3 and Rgt2, it is possible that additional proteins may function as glucose sensors.

Studies so far suggest there is a difference between *Cryptococcus* and *Saccharomyces* in glucose sensing and regulation. In *S. cerevisiae*, two glucose sensors, Snf3 and Rgt2, regulate other glucose transporter function via a SCF(Grr1) E3 ligase mediated UPS system. Our previous study has identified a Grr1 homolog in *C. neoformans*, Fbp1, which also functions as a SCF E3 ligase. However, expressing Fbp1 in a *grr1Δ* mutant failed to rescue the function of Grr1. Our preliminary studies also showed that some Grr1 substrate homologs in *C. neoformans* did not function as Fbp1 substrates, suggesting they may regulate different biological processes through different substrates (our unpublished data). In *S. cerevisiae*, Gpr1 functions as a glucose sensor to activate downstream Gpa2 G protein signaling that involves in cAMP levels. In *Cryptococcus*, although the G protein-cAMP signaling is overall conserved, its glucose receptor that activates this pathway remains unknown. We have identified one GPCR, Gpr4, that shares sequence and structure similarity with Gpr1, but it is not a glucose sensor. Compared with other yeasts, it is likely that *Cryptococcus* has developed a unique glucose sensing mechanism to accommodate its unique environmental and host conditions, which warrants further investigation.

## Materials and Methods

### Ethics Statement

The animal studies conducted at University of Medicine and Dentistry of New Jersey (UMDNJ) were in full compliance with all of the guidelines set forth by the Institutional Animal Care and Use Committee (IACUC) and in full compliance with the United States Animal Welfare Act (Public Law 98–198). The UMDNJ IACUCs approved all of the vertebrate studies. The studies were conducted in facilities accredited by the Association for Assessment and Accreditation of Laboratory Animal Care (AAALAC).

### Strains, Media, and Growth Conditions

*C. neoformans* and *S. cerevisiae* strains used in this study are listed in Table 1. Strains were grown at 30°C on yeast extract-peptone-dextrose (YPD) agar medium and synthetic (SD) medium. All other media were prepared as described previously [15].

**Table 1:** Strains used in this study.

*C. neoformans* strains	Genotype	Source/reference
H99	*MATα*	Perfect *et al.* (1993)
KN99**a**	*MAT* **a**	Nielsen *et al.* (2003)
CUX69	*MAT* **a** *hxs1::NEO*	This study
CUX146	*MATα hxs1::NEO*	This study
CUX145	*MAT* **a** *hxs1::NEO ura5*	This study
CUX148	*MAT* **a** *hxs1::NEO ura5 HXS1-URA5*	This study
S. cerevisiae strains		
YSB4742	*MATα his3Δ1, leu2Δ0, ura3Δ0*	ATCC yeast deletion collection
CEN.PK2-1C ( = VW1A)	*leu2-3,112 ura3-52 trp1-289 his3Δ1 MAL2-8c SUC2 hxt7Δ*	Wieczorke et al. (1999)
EBY.VW1000	*CEN.PK2-1C hxt13Δ::loxP hxt15Δ::loxP hxt16Δ::loxP hxt14Δ::loxP hxt12Δ::loxP hxt9Δ::loxP hxt11Δ::loxP hxt10Δ::loxP hxt8Δ::loxP hxt514Δ::loxP hxt2Δ::loxP*	Wieczorke et al. (1999)
YUX43	*EBY.VW1000 P_ADH1_-GFP*	This study
YUX44	*EBY.VW1000 P_ADH1_-GFP:HXS1*	This study
YUX45	*EBY.VW1000 _PADH1_-GFP:HXS2*	This study
YUX46	*EBY.VW1000 P_ADH1_-GFP:ScHXT1*	This study
YM6863	*MATalpha his3Δ1 leu2Δ0 ura3Δ0 MET15 lys2Δ0 rgt2::kanMX::natMX*	Mark Johnston
FM577	*MATa his3Δ1 leu2Δ0 ura3Δ0 met15Δ0 LYS2 snf3::kanMX*	Mark Johnston
YUX79	*MATalpha his3Δ1 leu2Δ0 ura3Δ0 MET15 lys2Δ0 rgt2::kanMX::natMX snf3::kanMX*	This study
YUX82	MATa his3*1 leu2*0 ura3*0 met15*0 LYS2 rgt2::kanMX::natMX snf3::kanMX P_ADH1_-GFP	This study
YUX83	MATa his3*1 leu2*0 ura3*0 met15*0 LYS2 rgt2::kanMX::natMX snf3::kanMX P_ADH1_-GFP:HXS1	This study
YUX84	MATa his3*1 leu2*0 ura3*0 met15*0 LYS2 rgt2::kanMX::natMX snf3::kanMX P_ADH1_-GFP:HXS2	This study
YUX85	MATa his3*1 leu2*0 ura3*0 met15*0 LYS2 rgt2::kanMX::natMX snf3::kanMX P_ADH1_-GFP:RGT2	This study

### Generation of *hxs1Δ* Mutants and their Complemented Strains

Mutants for *HXS1* and *HXS2* were generated in both H99 and KN99**a** strains background by overlap PCR as previously described. The 5′ and 3′ regions of the *HXS1* gene were amplified from H99 genomic DNA with primer pairs JH16929 and JH16930, and 16931 and 16932, respectively (see Table S1 for primer sequences). The 5′ and 3′ regions of the *HXS2* gene were amplified from H99 genomic DNA with primer pairs 16922 and 16923, and CX248 and 16925, respectively (see Table S1 for primer sequences). The dominant selectable markers (NEO^r^) were amplified with the M13 primers (M13F and M13R) from plasmid pJAF1. The dominant selectable markers (NAT^r^) were amplified with the M13 primers (M13F and M13R) from plasmid pPZP-NATcc. Each target gene replacement cassette was generated by overlap PCR with primers 16929 and 16932, 16922 and CX248. Purified overlap PCR products were precipitated onto 10 µl gold microcarrier beads (0.6 µm; Bio-Rad), and strains H99 or KN99**a** were biolistically transformed as described previously. Stable transformants were selected on YPD medium containing G418 (200 mg/L) or NAT (100 mg/L). To screen for mutants of *HXS1* or *HXS2* gene, diagnostic PCR was performed by analyzing the 5′ junction of the disrupted mutant alleles with primers 16935 and JH8994, 16928 and JH8994. Positive transformants were identified by PCR screening with primers 16933 and 16934, 16922 and 16927, respectively. While *hxs1Δ* null mutants were isolated, generation of *hxs2Δ* deletion mutants was not successful despite extensive effort.

To generate complemented strains of the *hxs1Δ* mutant, *ura5* mutant strains were generated by selecting colonies grown on agar plates containing 0.1% 5-fluoroorotic acid (5-FOA). A genomic DNA fragment that contains a 1.5-kb upstream promoter region, the *HXS1* open reading frame (ORF), and its 500-bp downstream region was amplified in a PCR using primers CX213 and CX214. This PCR fragment was digested with XmaI and EcoRI and cloned into the vector pJAF7 containing *URA5* selective marker gene. The *HXS1-URA5* construct was used to biolistically transform in a *hxs1Δ* mutant strain. The ectopic expression of the *HXS1* gene was confirmed by RT-PCR. Phenotypic assays were performed to identify transformants in which the *hxs1Δ* phenotype was complemented.

### Detection of Gene Expression Using Quantitative Reverse Transcription-PCR (RT-PCR)

The expression of *HXT1* and *HXT2* genes was measured at mRNA level via quantitative real-time PCR (qRT-PCR) in strains grown with different concentration of glucose. Cultures of *C. neoformans* var. *grubii* wild-type strain H99 and *hxs1Δ* were grown overnight on YPG (2% galactose) liquid medium at 30°C with shaking. Collected cells were washed with distilled H_2_O (dH_2_O), resuspended in YP containing 0.1% glucose and YP containing 2% glucose medium and incubated for 2 hrs. Total RNAs were prepared from cells with each treatment and cDNA was synthesized as described below.

We measured *HXS1* and *HXS2* mRNA levels via quantitative real-time PCR (qRT-PCR) in cells grown with or without glucose. Cultures of *C. neoformans* var. *grubii* wild-type strain H99 were grown overnight on YPD (2% glucose) or YPG (2% galactose) liquid medium at 30°C with shaking. Collected cells were washed with distilled H_2_O (dH_2_O), resuspended in YPD or YPG medium and incubated for 2 h. Cells were then collected and washed with dH_2_O. Cells collected from YPD were resuspended in YP containing 0.1% glucose or YPG, while cells collected from YPG were resuspended in YP containing 0.1% glucose or YPD. Both cultures were incubated for 2 hrs. Total RNAs were prepared from cells with each treatment.

Purified RNAs were quantified using a Nanodrop spectrometer (Thermo Scientific) and were used as templates for PCR amplification with primers of glyceraldehyde-3-phosphate dehydrogenase gene *(GAPDH)* to determine potential genomic DNA contamination. First strand cDNAs were synthesized using a Superscript III cDNA synthesis kit (Invitrogen) following the manufacturer’s instructions. Expression levels of *HXT1, HXT2, SUC2,* and *GAPDH* were analyzed using SYBR advantage QPCR premix reagents (Clontech) with an Mx4000 QPCR system (Stratagene) as previously described [37], [38]. Gene-expression levels were normalized using the endogenous control gene *GAPDH*, and the relative levels were determined using the comparative C_T_ method.

### Assays for Virulence Factors

Assays for melanin and capsule production were performed as previously described [28]. In brief, melanin production was tested on L-DOPA medium, and incubated at 30°C or 37°C for three days and pigmentation of fungal colonies was assessed and photographed. Capsule production was induced on Dulbecco Modified Eagle’s (DME) agar medium and incubated at 37°C for three days. Capsule size was visualized by India ink staining and observed with an Olympus CX41 microscope.

### Assays for Stress Responses and Cell Integrity

Each strain was incubated overnight at 30°C in YPD and sub-cultured in fresh YPD medium to OD_600_ ∼0.7. The cells were washed, resuspended, and serially diluted (1∶10) in dH_2_O and spotted (5 µl) on YPD agar plates containing 1.5 M NaCl, or 1.0 M KCl for osmotic shock, or 2.5 mM and 5.0 mM H_2_O_2_ for oxidative stress. To test cell integrity, cells were also spotted on YPD agar plates containing 0.05% SDS, 0.5% Congo Red, or 20 µg/ml Calcofluor White (CFW). Plates were incubated at both 30°C and 37°C for two days and photographed.

For growth assay on media with different carbon source, Cultures of wild type H99, the *hxs1Δ* mutant and its complemented strain were inoculated on YPD for 20 hr. Cells were washed and 1×10^5^ cells of each strain were inoculated into the wells of 96-well plates containing 100 µl YP supplemented with different carbon source. The plates were kept in a PerkinElmer precisely Envision 2014 Multilabel Reader and incubated at 30°C with shaking (350RPM) and OD600 were measured in real time every half hour.

### Glucose Uptake Assay

Full-length cDNAs of the *HXS1*, *HXS2* genes were amplified from *C. neoformans* H99 total cDNA and were cloned into the yeast expression vector pTH74 to generate GFP fusion constructs, under the control of the *ADH1* promoter. *HXS1* and *HXS2* expression plasmids were introduced into an *S. cerevisiae* strain EBY.VW1000 that lacks all 20 *HXT* transporters [30]. The expression of *Cryptococcus HXS1* or *HXS2* in this yeast heterologous system was verified by both GFP localization and RT-PCR using gene-specific primers. Yeast strains were tested for growth on different medium at 30°C.

The *S. cerevisiae* control strain CEN.PK2.1C, the *S. cerevisiae hxtΔ* mutant strain EBY.VW1000, and EBY.VW1000 expressing empty vector, *GFP*-*HXS1* or *GFP*-*HXS2* genes from *C. neoformans* were grown in YP containing 2% maltose liquid cultures overnight at 30°C. Collected cells were washed with dH_2_O, resuspended in YP containing 2% maltose, and incubated for 2 hrs. Then cells were suspended in PBS at a final concentration of 2×10^8^ cells/ml for uptake assay. Each 100 µl cell suspension was mixed with 100 µl labeled glucose (^3^H-glucose) solution at room temperature. Samples (100 µl) were removed after 30 s, 1 min, 5 min, and 10 min, and mixed with 1 ml ice-cold water to stop the reactions. Cells were immediately collected on fiber filters, washed three times with 10 ml of ice-cold water, and transferred to scintillation vials for measurement.

### Virulence Studies

Survival curves of infected mice in a *Cryptococcus* murine inhalation model as previously described [38]. Female A/Jcr mice (NCI-Frederick) were inoculated intranasally with the following strains: H99, the *hxs1Δ* mutant and its complemented strain. Groups of ten mice were infected with 1×10^5^ yeast cells for each strain. Over the course of the experiments, animals that appeared moribund or in pain were sacrificed by CO_2_ inhalation. Survival data from the murine experiments were statistically analyzed between paired groups using the long-rank test of the PRISM program 4.0 (GraphPad Software, San Diego, CA). P values of <0.001 were considered significant. Infected animals were sacrificed at the endpoint of the experiment according to the UMDNJ IACUC-approved animal protocol.

## Supporting Information

Figure S1 **Phylogram of hexose transporter homologs in** ***S. cerevisiae, C. albicans*** **, and** ***C. neoformans.*** The phylogenetic tree was generated using ClustalX 2.0 program and viewed using the TreeView software. A cluster of proteins showed high sequence identity was highlighted.
(TIF)

Figure S2 **Expression of the** ***HXS1*** **and** ***HXS2*** **under different glucose conditions.** *C. neoformans* wild type H99 was cultured on YPD (2% glucose) or YPG (0% glucose), or cultured on medium containing 2% glucose (YPD) and switched to 0.1% glucose (YP0.1D) or 0% glucose (YPG) and incubated for 2 more hrs. RNAs were extracted and cDNAs synthesized from those cells and were used as templates for qRT-PCR. PCR products amplified for 35 cycles were loaded on 1% agarose gel and photographed. GAPDH gene was used as an internal control.
(TIF)

Table S1 **Primers used in this study.**
(DOC)
