# Supplementary figures and images for: The Glucose Sensor-Like Protein Hxs1 Is a High-Affinity Glucose Transporter and Required for Virulence in Cryptococcus neoformans
Source: PLoS One. 2013 May 14;8(5):e64239. doi: 10.1371/journal.pone.0064239 (PMC3653957; doi:10.1371/journal.pone.0064239)

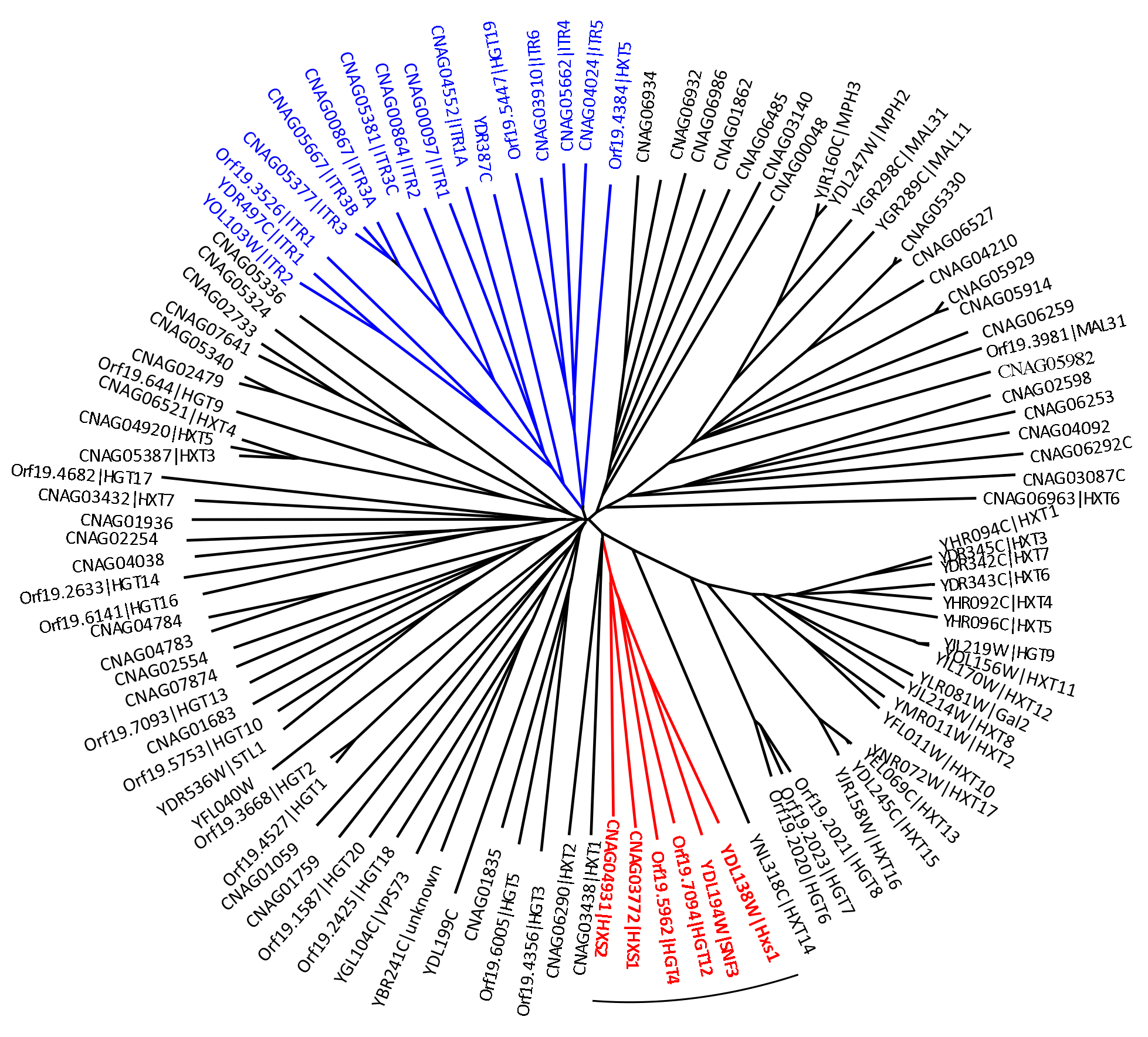

Supplement: Figure S1 — Phylogram of hexose transporter homologs in S. cerevisiae, C. albicans , and C. neoformans. The phylogenetic tree was generated using ClustalX 2.0 program and viewed using the TreeView software. A cluster of proteins showed high sequence identity was highlighted. (TIF) [file pone.0064239.s001.tif]

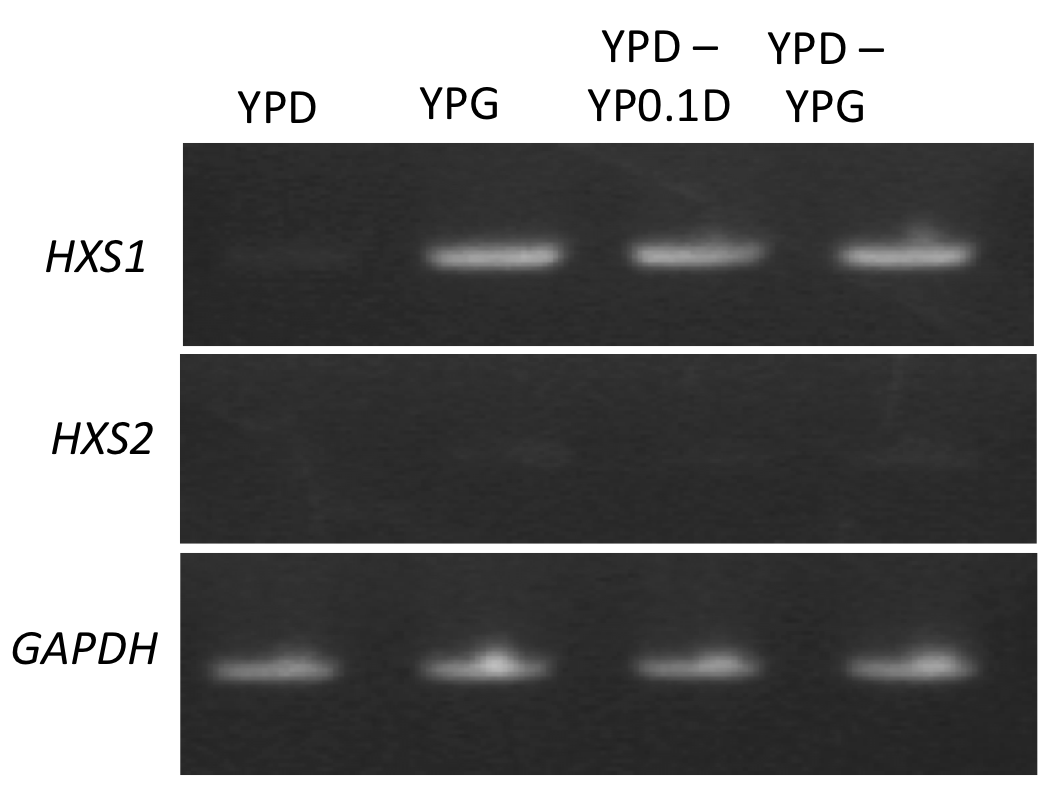

Supplement: Figure S2 — Expression of the HXS1 and HXS2 under different glucose conditions. C. neoformans wild type H99 was cultured on YPD (2% glucose) or YPG (0% glucose), or cultured on medium containing 2% glucose (YPD) and switched to 0.1% glucose (YP0.1D) or 0% glucose (YPG) and incubated for 2 more hrs. RNAs were extracted and cDNAs synthesized from those cells and were used as templates for qRT-PCR. PCR products amplified for 35 cycles were loaded on 1% agarose gel and photographed. GAPDH gene was used as an internal control. (TIF) [file pone.0064239.s002.tif]
