# Supplementary material for: The Glucose Sensor-Like Protein Hxs1 Is a High-Affinity Glucose Transporter and Required for Virulence in Cryptococcus neoformans
Source: PLoS One. 2013 May 14;8(5):e64239. doi: 10.1371/journal.pone.0064239 (PMC3653957; doi:10.1371/journal.pone.0064239)
Supplement: Table S1 — Primers used in this study. (DOC) [file pone.0064239.s003.doc]

Table S1 Primers used in this study

| Primers | Sequences(5’-3’) | References/Note |
| --- | --- | --- |
| CX5 | GTAAAACGACGGCCAG | M13F |
| CX6 | CAGGAAACAGCTATGAC | M13R |
| CX49 | TGA GAA GGA CCC TGC CAA CA | GAPDH qRT-PCR |
| CX50 | ACT CCG GCT TGT AGG CAT CAA | GAPDH qRT-PCR |
| CX221 | TAGCTGCGTGTGTGGTTTACGCTA | HXS2 qRT-PCR |
| CX222 | ACAGCTTGACTGTTCTCGGCTTCA | HXS2 qRT-PCR |
| CX223 | ATGCAGCAATGGACTGGTGTCAAC | HXS1 qRT-PCR |
| CX224 | TGAACTCGCAGATGAGCATACCGA | HXS1 qRT-PCR |
| CX340 | AGTCACGTCGGTTTCCTTGGTCTT | SUC2 qRT-PCR |
| CX341 | TCGTCCTTCCCGAAGTTTGTGAGT | SUC2 qRT-PCR |
| CX460 | AGGAAAATGAGGGTGGAAAGG | HXT1 qRT-PCR |
| CX461 | GAGAATGGCTTGGATAGAGTACG | HXT1 qRT-PCR |
| CX462 | TGTCCTCATTGCCTTTACCTG | HXT2 qRT-PCR |
| CX463 | TGAGGTTGAAGAGCCAGTTG | HXT2 qRT-PCR |
| 16929 | CCCGCAAAACGTATGCATAGG | HXS1 KO left |
| 16930 | CTGGCCGTCGTTTTACGGAAAGAACAAGAGCTCGAAG | HXS1 KO left |
| 16931 | GTCATAGCTGTTTCCTGAGTCAGAGTTGGTGGAGCAAG | HXS1 KO right |
| 16932 | TGTTGAGCCGGAATACATGAC | HXS1 KO right |
| 16933 | GCGACTGCCACCCTTCTCAAC | HXS1 KO negative |
| 16934 | ACGGAATGGAAAGGCACTTAC | HXS1 KO negative |
| 16935 | GTAGGAGTAGTGAGGTATCAAG | HXS1 KO positive |
| CX213 | ATACGGCAAGGCTTGCCTGAC | HXS1 complementation |
| CX214 | GCAACTGGCCGTCGTTTTACCTGCGCTACTGCACGAGCAAG | HXS1 complementation |
| CX385 | GAATTCCCGGGGATCCGAATGCACTTGCCTGGTGGTGC | HXS1 Infusion cloning and for yeast expression |
| CX386 | GCAGGTCGACGGATCCTTAAACTTGCTCCACCAACTC | HXS1 Infusion cloning and for yeast expression |
| CX389 | GAATTCCCGGGGATCCGAATGAATTCAACTCCCGATCTA | ScHXT1 Infusion cloning and for yeast expression |
| CX390 | GCAGGTCGACGGATCCTTATTTCCTGCTAAACAAACTC | ScHXT1 Infusion cloning and for yeast expression |
| CX391 | GAATTCCCGGGGATCCAGATGCCCCTTCCAAAGTTAGC | HXS2 Infusion cloning and for yeast expression |
| CX392 | GCAGGTCGACGGATCCTCATTTCAAGAACTCCAACAT | HXS2 Infusion cloning and for yeast expression |
| CX491 | GTTAACAAATGCCCACCTGAC | Sc HXT1 qRT-PCR |
| CX492 | TCACTTAAACCGACAGCCTG | Sc HXT1 qRT-PCR |
| CX493 | CAAACTTCTATCCACTCTACTCCG | Sc HXT2 qRT-PCR |
| CX494 | CAACCAAAGACAAACCCACCA | Sc HXT2 qRT-PCR |
| JH8994 | TGTGGATGCTGGCGGAGGATA | For positive screen |
| 16922 | CCTTCGTCAGAACCAAAGTTG | HXS2 KO left |
| 16923 | CTGGCCGTCGTTTTACTGATCGGTGCCCCACGTTGAC | HXS2 KO left |
| CX248 | GTCATAGCTGTTTCCTGGGAGCTTTCGCTTCTTTCTCTG | HXS2 KO right |
| 16925 | TAGGGGATTAGGAGCTAGGTC | HXS2 KO right |
| 16926 | AGCTCGGGTAGAGACTCCTTG | HXS2 KO negative |
| 16927 | CAGTCAATTGCTGTAAGGCTTG | HXS2 KO negative |
| 16928 | GAGTGATTCCAGGGCTGTGATG | HXS2 KO positive |
|  |  |  |
